# Morphological and Functional Relationship Between OCTA and FA/ICGA Quantitative Features in AMD-Related Macular Neovascularization

**DOI:** 10.3389/fmed.2021.758668

**Published:** 2021-10-20

**Authors:** Alessandro Arrigo, Emanuela Aragona, Alessandro Bordato, Alessia Amato, Federico Borghesan, Francesco Bandello, Maurizio Battaglia Parodi

**Affiliations:** Department of Ophthalmology, San Raffaele Scientific Instititute, Milan, Italy

**Keywords:** age-related macular degeneration, OCT, OCTA, MNV, vessel density, vessel tortuosity

## Abstract

**Background:** The aim was to study the relationship between quantitative information provided by optical coherence tomography (OCT) angiography (OCTA) and conventional angiography in macular neovascularization (MNV) secondary to age-related macular degeneration (AMD).

**Methods:** The research was designed as an interventional, prospective study. We included 66 eyes (66 patients) affected by naïve MNV. Multimodal imaging included structural OCT, OCTA, fluorescein angiography (FA), and indocyanine green angiography (ICGA). The follow-up lasted 1 year. Patients were treated by PRN anti-VEGF injections. Based on FA/ICGA examinations, we divided the patients into two categories: low vessel tortuosity (VT) (<8.40) and high VT (>8.40), correlating VT with the MNV area, leakage area, speckled fluorescence (SF) quadrants and MNV area/leakage area ratio.

**Results:** Mean baseline BCVA was 0.50 ± 0.61 LogMAR, improved to 0.31 ± 0.29 LogMAR after 1 year (*p* < 0.01), with a mean number of 7 ± 2 anti-VEGF injections. The patients revealed type-1 MNV in 36 eyes (55%), mixed type 1 and 2 MNV in 18 eyes (27%), and type-2 MNV in 12 eyes (18%). MNV eyes in high-VT MNV featured poorer BCVA, CMT, and OCTA parameters, higher SF quadrants, and less exudation, compared with low-VT MNV (*p* < 0.01). Moreover, 30% of high-VT MNV eyes developed outer retinal atrophy.

**Conclusions:** Low VT MNV turned out to be more exudative at the baseline but less damaging to the outer retinal structures, whereas high VT MNV proved to be less exudative but more prone to lead to atrophic changes and visual function deterioration. VT may be usefully applied to artificial intelligence-based models designed to characterize MNV secondary to AMD.

## Synopsis

Two clinically different MNV subforms can be identified by combining OCTA and conventional angiography: an MNV that is characterized by greater exudation but is less damaging to the outer retinal structures, and an MNV that is less exudative but leads to irreversible anatomical impairment.

## Introduction

Macular neovascularization and exudation are possible complications encountered in the advanced stages of age-related macular degeneration (AMD) (1), causing a progressive deterioration of the visual function. Anti-VEGF intravitreal injections represent the gold standard for the treatment of the condition and have radically changed its natural history and the outcome for patients with AMD (2). However, the response to anti-VEGF treatment is extremely variable, thus rendering the outcome unpredictable. Nowadays, optical coherence tomography (OCT) angiography (OCTA) represents an extremely powerful non-invasive approach to the detailed analysis of MNV secondary to AMD (3–5). Quantitative OCTA parameters, in the first instance, MNV vessel tortuosity (VT), have recently been proposed to differentiate clinically different MNV subgroups (6). This quantitatively based categorization of the MNV lesions depended on the perfusion features of the MNV and its clinical activity and proved to be less influenced by the type of MNV (6, 7).

The strength of this new quantitative approach is that it offers an estimate of MNV activity, thus providing useful information about the evolution of the neovascular lesion and the damage caused to retinal structures. However, from the point of view of its association with the functional features of the MNV, the methodology fails to provide an assessment of the relationship between the information supplied by OCTA and data obtained from dye-based angiography. In particular, OCTA is well-known to yield little regarding the blood-retinal barrier breakdown and the exudative phenomena, which are, in contrast, well detected by dye-based angiography.

The aim of the present paper was to classify the AMD-related MNV by combining MNV VT on OCTA with findings obtained by standard dye angiography.

## Methods

The study was designed as a prospective, interventional case series. Consecutive patients with AMD newly diagnosed with MNV were recruited at the Ophthalmology Unit of San Raffaele Hospital, Milan, Italy, from January 2018 and December 2019, and followed for 1 year. The study was approved by the Ethical Committee of the Vita-Salute San Raffaele University in Milan (protocol ID: MIRD) and conducted in accordance with the Declaration of Helsinki. All MNV patients underwent Ranibizumab.5-mg intravitreal injections, starting with three monthly injections, followed by further treatments in accordance with a *pro re nata* regimen. The inclusion criteria were: naïve AMD-related MNV; classified as type 1, type 2, and mixed type 1 and 2 on the basis of fluorescein angiography (FA) and indocyanine green angiography (ICGA) (Spectralis HRA+OCT; Heidelberg Engineering, Heidelberg, Germany). We excluded polypoidal choroidal vasculopathy and retinal angiomatous proliferation because of difficulties in achieving a reliable identification of the neovascular lesion on OCTA. Further exclusion criteria were: high media opacities, any other ophthalmological disorder, ophthalmological surgery within the last 6 months, and any systemic condition potentially affecting the analyses.

Ophthalmological examination included BCVA measurement using standard ETDRS charts, slit lamp biomicroscopy of anterior and posterior segments, and Goldmann applanation tonometry. Structural OCT acquisitions included raster, radial, and dense scans with a high number of frames (ART > 25) and enhanced depth imaging (EDI). Structural OCT data were used to measure central macular thickness (CMT) and exudation thickness at the baseline and at the 1-year follow-up. OCTA images were obtained using a swept source OCT DRI Topcon Triton (Topcon Corporation, Tokyo, Japan). OCTA scans included high-resolution 3 × 3 and 4.5 × 4.5 mm acquisitions. Only high-quality images, evaluated by Topcon Imaging Quality factor > 70, were considered. Two expert readers (AA and EA) carefully inspected and eventually manually corrected all OCTA segmentations to address for possible residual artifacts.

We isolated superficial (SCP), deep (DCP), and choriocapillaris (CC) plexa from 4.5 × 4.5 mm scans, as well as the MNV network from 3 × 3 mm high-resolution OCTA reconstructions. To obtain vascular plexa vessel density (VD) values and MNV VT values, we performed the same post-processing steps already described in previous papers (6–10). All these parameters were calculated by means of ImageJ software (11). The first step in our post-processing analyses was to binarize OCTA reconstructions using the following pipeline: Import.tiff image -> Adjust -> Threshold -> Automatic threshold -> Mean thresholding -> Export binarized image. VD was obtained by calculating the ratio of white to black pixels, placing the manually segmented foveal avascular zone among the exclusion criteria. VT is defined as the ratio of the shortest pathway to the straight-line length (12), and provides information about the vascular perfusion (6, 7, 13, 14). MNV VT was calculated through the following pipeline: Loading binarized MNV image -> Skeletonize -> Analyze skeleton -> Euclidean distance measurement.

Optical coherence tomography angiography quantitative analyses were carried out on the basis of baseline reconstructions alone.

We applied an MNV VT cutoff of 8.40 to differentiate two clinically significant MNV subforms: low-VT MNV (VT <8.40) and high-VT MNV (VT > 8.40) (6, 7). We performed a fresh ROC analysis to confirm the validity of this previously proposed MNV VT cutoff (6, 7), as well as to determine the presence of any further MNV VT cutoff value.

The following parameters were considered: leakage area (defined as the extension of the region affected by leakage 5 min after the fluorescein dye injection), MNV area (measured on ICGA), and speckled fluorescence (SF) (defined as multiple punctate spots of hyperfluorescence observed 5 min after the fluorescein dye injection). SF was analyzed by dividing the posterior pole into four quadrants (two temporal and two nasal) and by considering only the quadrants involved, using the center of the MNV as a reference point. These metrics were obtained by the measurement tools provided by Heidelberg software. Furthermore, we calculated an MNV area/leakage area ratio so as to quantify the amount of leakage in relation to the size of the MNV and to obtain a parameter associated with the activity of the MNV. The analyses were conducted by two expert graders (AA and EA) at least two times in order to calculate the reproducibility and repeatability of the measurements. The intraclass correlation coefficient (ICC) was calculated to evaluate the agreement between the two operators.

The aim of the present paper was to classify the AMD-related MNV by combining MNV VT on OCTA with findings obtained by standard dye angiography.

The statistical analyses were performed using the SPSS software package (SPSS, Chicago, Illinois, USA). We considered age, sex, and number of injections as fixed factors for the analysis. The sample size was calculated on the basis of similar studies present in the literature, considering a minimum of 50 eyes needed to reject the null hypothesis with a confidence interval of 95% and a margin of error of 5%. Continuous variables were analyzed through the two-tailed *t*-test. We stratified MNV eyes according to the MNV VT value and assessed possible intergroup statistical differences. We also evaluated the possible influence of the type of MNV (type 1, type 2, and mixed) on the quantitative investigation, as well as possible intergroup differences, through one-way ANOVA analysis, with Bonferroni correction for multiple comparisons. In view of the inclusion of 11 different metrics, the statistical significance was set at *p* < (0.05/11 = 0.0045). The Tau-Kendall correlation analysis (SPSS, Chicago, Illinois, USA) was used to assess the relationship between all the parameters reviewed. In this case, we considered a *p* < 0.01 to be statistically significant.

## Results

We collected data from 80 eyes of 80 patients affected by naïve AMD-related MNV. Fourteen eyes were excluded, owing to high media opacities. The remaining 66 eyes of 66 patients with MNV (34 males; mean age 78 ± 8 years) were included in the study and completed the entire follow-up. Mean BCVA was 0.50 ± 0.61 LogMAR at the baseline, improving to 0.31 ± 0.29 LogMAR after the 1-year follow-up (*p* = 0.002), with a mean number of 7 ± 2 injections administered. CMT was 408 ± 105 μm at the baseline, improving to 348 ± 76 μm after 1 year (*p* =.003). In accordance with MNV angiographic classification, we found 36 eyes with type 1 (55%), 18 eyes with mixed types 1 and 2 (27%), and 12 with type 2 (18%) MNV. As reported in Table 1, type-2 MNV showed higher exudation, as indicated by the significantly lower MNV/leakage ratio, and greater CMT. The mixed MNV type displayed intermediate values, compared with type 1 and type 2 lesions. Interestingly, the number of intravitreal injections administered was similar among the three MNV subtypes. Moreover, MNV VT values were similar in the entire cohort of type 1, type 2, and mixed lesions.

**Table 1:** Clinical and imaging data in MNV types.

**Clinical and Imaging data in MNV Types**						
**Parameter**	**MNV type**	**Number**	**Mean** **±** **STD**	***p*** **Value**		
				**1 vs. 2**	**1 vs. 3**	**2 vs. 3**
Age	Type 1	1	77 ± 7	*p =* 0.158	*p =* 0.934	*p =* 0.142
	Mixed	2	80 ± 8			
	Type 2	3	75 ± 7			
Speckled fluorescence (quadrants)	Type 1	1	1.3 ± 1	*p =* 0.242	*p =* 0.745	*p =* 0.978
	Mixed	2	1 ± 1			
	Type 2	3	1 ± 1			
Leakage area (μm^2^)	Type 1	1	4.4 ± 3.5	*p =* 0.838	*p =* 0.544	*p =* 0.768
	Mixed	2	3.8 ± 5.4			
	Type 2	3	4.2 ± 4.1			
MNV area	Type 1	1	3.5 ± 3.3	*p =* 0.964	*p =* 0.225	*p =* 0.452
	Mixed	2	2.6 ± 3.1			
	Type 2	3	1.5 ± 1.6			
MNV/leakage ratio	Type 1	1	0.8 ± 0.3	*p =* 0.03	*p =* 0.002*	*p =* 0.714
	Mixed	2	0.6 ± 0.3			
	Type 2	3	0.5 ± 0.2			
LogMAR BCVA baseline	Type 1	1	0.46 ± 0.60	*p =* 0.845	*p =* 0.698	*p =* 0.751
	Mixed	2	0.63 ± 0.78			
	Type 2	3	0.44 ± 0.27			
LogMAR BCVA 1-year	Type 1	1	0.29 ± 0.28	*p =* 0.641	*p =* 0.565	*p =* 0.742
	Mixed	2	0.35 ± 0.27			
	Type 2	3	0.29 ± 0.34			
CMT baseline (μm)	Type 1	1	371 ± 77	*p =* 0.227	*p* < 0.001*	*p =* 0.005*
	Mixed	2	420 ± 91			
	Type 2	3	503 ± 137			
CMT 1-year (μm)	Type 1	1	331 ± 73	*p =* 0.478	*p =* 0.262	*p =* 0.411
	Mixed	2	368 ± 89			
	Type 2	3	370 ± 58			
N. Intravitreal injections	Type 1	1	6 ± 2	*p =* 0.888	*p =* 0.956	*p =* 0.961
	Mixed	2	7 ± 2			
	Type 2	3	7 ± 2			
VT MNV	Type 1	1	8.5 ± 1.40	*p =* 0.756	*p =* 0.842	*p =* 0.798
	Mixed	2	8.5 ± 1.3			
	Type 2	3	8.1 ± 0.7			
VD SCP	Type 1	1	0.38 ± 0.02	*p =* 0.545	*p =* 0.651	*p =* 0.498
	Mixed	2	0.37 ± 0.02			
	Type 2	3	0.38 ± 0.02			
VD DCP	Type 1	1	0.36 ± 0.02	*p =* 0.631	*p =* 0.492	*p =* 0.468
	Mixed	2	0.36 ± 0.03			
	Type 2	3	0.37 ± 0.02			
VD CC	Type 1	1	0.45 ± 0.03	*p =* 0.825	*p =* 0.618	*p =* 0.522
	Mixed	2	0.45 ± 0.03			
	Type 2	3	0.47 ± 0.02			

MNV, macular neovascularization; BCVA, best-corrected visual acuity; CMT, central macular thickness; VT, vessel tortuosity; VD, vessel density; SCP, superficial capillary plexus; DCP, deep capillary plexus; CC, choriocapillaris. Statistically significant values are marked by asterisks (*).

**Figure 1:**
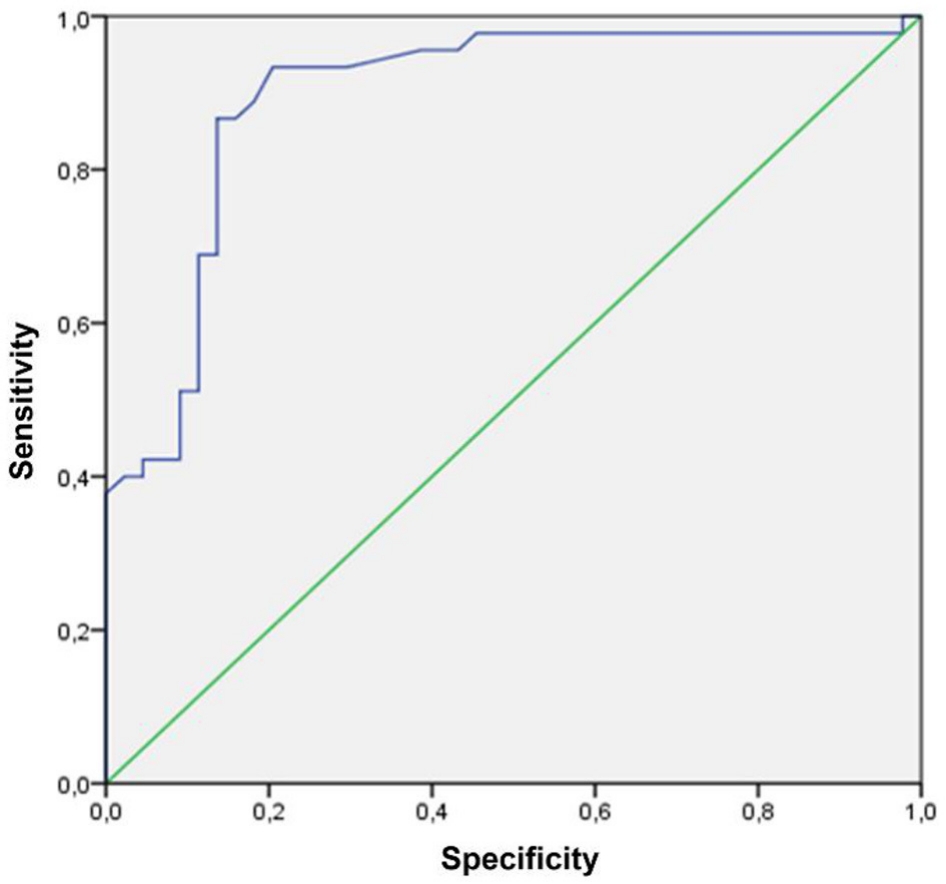
An ROC curve of an MNV VT cutoff (VT cutoff value 8.40; sensitivity, 0.90; specificity, 0.92).

**Figure 2:**
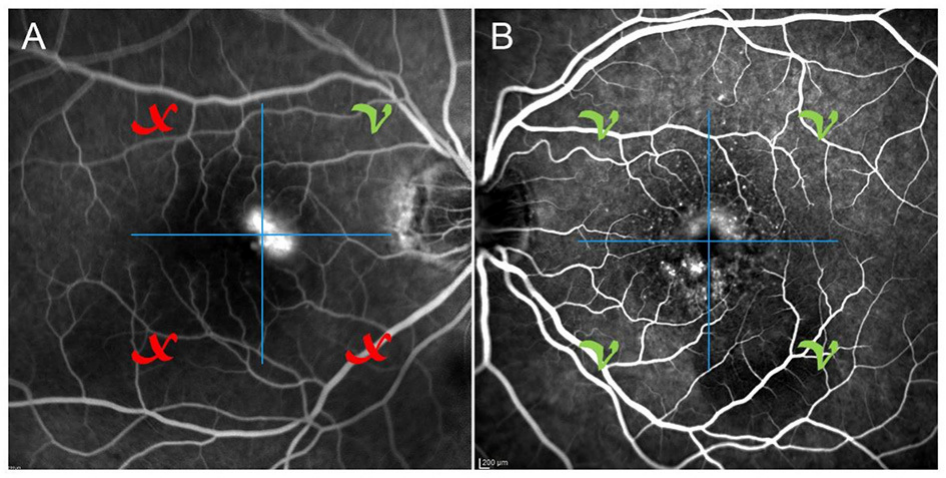
One case of poorly involved SF quadrants **(A)** on fluorescein angiography and one case of totally involved SF quadrants **(B)** on fluorescein angiography.

**Figure 3:**
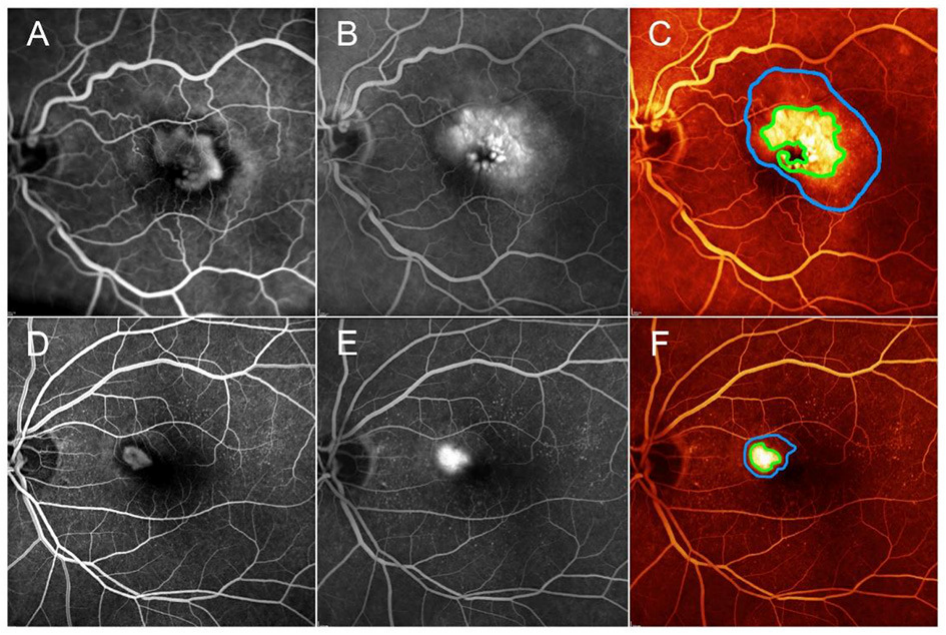
One case of low-MNV/leakage ratio, corresponding to high exudation area compared with MNV lesion detected on fluorescein angiography **(A,B)**. MNV area and leakage area are marked by green and blue lines, respectively **(C)**. One case of high-MNV/leakage ratio, corresponding to a lower exudation area, compared with MNV lesion detected on fluorescein angiography **(D,E)**. MNV area and leakage area are marked by green and blue lines, respectively **(F)**. Green V and red X correspond to the quadrants showing SF or quadrants without SF, as detected by FA, respectively.

Categorizing MNV on the basis of a VT cutoff value of 8.40, confirmed by the new ROC analysis (sensitivity, 0.90; specificity, 0.92) (Figure 1), allowed us to distinguish two different MNV subforms, namely low-VT MNV (41 eyes; VT <8.40) and high-VT MNV (25 eyes; VT > 8.40). Specifically, eyes in the high-VT MNV group showed worse final BCVA (0.24 ± 0.24 vs. 0.43 ± 0.32), final CMT and baseline DCP VD (*p* < 0.001) (Table 2). Moreover, 30% of eyes in the high-VT MNV group developed macular atrophy (understood as complete retinal pigment epithelium and outer retinal atrophy), as against no eye in the low-VT MNV group. Low-VT MNV showed less SF quadrant involvement than high-VT MNV (Figure 2) (*p* < 0.001), whereas the MNV and leakage areas were similar (*p* = 0.22 and *p* = 0.33, respectively) (Table 2). By contrast, the MNV/leakage ratio proved significantly higher in cases of high-VT MNV than in low-VT MNV (*p* < 0.001) (Figure 3). MNV VT values turned out to be stable over the entire follow-up (*p* = 0.452). The correlation analysis revealed that high-VT MNV is characterized by more SF and less exudation, expressed both as a high MNV area/leakage area ratio and thinner CMT. The MNV and leakage areas correlated significantly with worse VD of the CC at the baseline. Indicators of lower exudation (higher MNV/leakage ratio and CMT) correlated significantly with higher MNV VT and lower DCP VD (all correlation data are listed in Table 3). Table 4 summarizes the most important characteristics of low- and high-VT MNV.

**Table 2:** Quantitative analysis in functionally different MNV subgroups (VT cutoff 8.40).

**Quantitative analysis in functionally different MNV subgroups (VT cutoff 8.40)**			
**Parameter**	**MNV Subgroups**	**Mean ± STD**	***p***
Age	Low VT MNV	78 ± 8	0.88
	High VT MNV	78 ± 8	
Speckled fluorescence (quadrants)	Low VT MNV	1.05 ± 1.03	<0.001*
	High VT MNV	1.63 ± 0.97	
Leakage area (μm^2^)	Low VT MNV	3.78 ± 3.32	0.33
	High VT MNV	4.84 ± 5.38	
MNV area (μm^2^)	Low VT MNV	2.49 ± 2.91	0.22
	High VT MNV	3.55 ± 4.13	
MNV/leakage ratio	Low VT MNV	0.59 ± 0.31	<0.001*
	High VT MNV	0.88 ± 0.24	
MNV Type (Type 1/Type 2/Mixed)	Low VT MNV	22/10/9	=0.33
	High VT MNV	14/8/3	
LogMAR BCVA baseline	Low VT MNV	0.63 ± 0.71	<0.001*
	High VT MNV	0.28 ± 0.25	
LogMAR BCVA 1-year	Low VT MNV	0.24 ± 0.24	<0.001*
	High VT MNV	0.43 ± 0.32	
CMT baseline (μm)	Low VT MNV	484 ± 88	<0.001*
	High VT MNV	380 ± 127	
CMT 1-year (μm)	Low VT MNV	362 ± 77	0.03
	High VT MNV	324 ± 70	
N. Intravitreal injections	Low VT MNV	7.2 ± 1.5	<0.001*
	High VT MNV	5.2 ± 1.9	
VT MNV	Low VT MNV	7.6 ± 0.4	<0.001*
	High VT MNV	9.6 ± 1.4	
VD SCP	Low VT MNV	0.37 ± 0.02	0.18
	High VT MNV	0.38 ± 0.03	
VD DCP	Low VT MNV	0.37 ± 0.02	<0.001*
	High VT MNV	0.34 ± 0.02	
VD CC	Low VT MNV	0.45 ± 0.03	0.66
	High VT MNV	0.45 ± 0.03	

*MNVs were divided in accordance with a VT cutoff value of 8.40*.

*MNV, macular neovascularization; BCVA, best-corrected visual acuity; CMT, central macular thickness; VT, vessel tortuosity; VD, vessel density; SCP, superficial capillary plexus; DCP, deep capillary plexus; CC, choriocapillaris*.

Statistically significant values are marked by asterisks (*).

**Table 3:** Correlation analysis in functionally different MNV subgroups (VT cutoff 8.40).

**Low-high MNV VT subgroups**	**Parameter**	**Speckled fluorescence (quadrants)**	**MNV area/leakage area ratio**	**Baseline BCVA**	**Final BCVA**	**Intravitreal injection number**
	Tau Kendall coeff.	0.356	0.299	−0.322	0.290	−0.449
	*p*	<0.001	=0.003	=0.002	=0.006	<0.001
Low-high MNV VT subgroups	**Parameter**	**MNV VT**	**Baseline CMT**	**Final CMT**	**VD DCP**	
	Tau Kendall coeff.	0.700	−0.343	−0.291	−0.283	
	*p*	<0.001	<0.008	=0.01	=0.005	
MNV type (Type 1, Mixed, Type 2)	**Parameter**	**MNV area**	**MNV area/leakage area ratio**	**Baseline CMT**	**Final CMT**	
	Tau Kendall coeff.	−0.319	−0.373	0.377	0.273	
	*p*	=0.004	=0.002	<0.001	=0.006	
Leakage area	**Parameter**	**MNV Area**	**VD CC**			
	Tau Kendall coeff.	0.705	−0.281			
	*p*	<0.001	=0.003			
MNV area	**Parameter**	**VD CC**				
	Tau Kendall coeff.	−0.250				
	*p*	=0.003				
MNV area/leakage area ratio	**Parameter**	**Intravitreal injection number**	**MNV VT**	**Baseline CMT**	**Final CMT**	**VD DCP**
	Tau Kendall coeff.	−0.337	0.346	−0.336	−0.291	−0.257
	*p*	=0.008	<0.001	<0.001	=0.004	=0.005
MNV VT	**Parameter**	**Baseline exudation**	**Baseline CMT**	**Final CMT**	**VD DCP**	
	Tau Kendall coeff.	−0.326	−0.256	−0.303	−0.297	
	*p*	=0.009	=0.002	=0.004	=0.005	
VD DCP	**Parameter**	**Speckled fluorescence (quadrants)**	**Baseline exudation**	**Final exudation**	**Baseline CMT**	**Final CMT**
	Tau Kendall coeff.	−0.241	0.198	0.315	0.246	0.296
	*p*	=0.004	=0.008	=0.005	=0.004	=0.004

*MNVs were divided in accordance with a VT cutoff value of 8.40. MNV, macular neovascularization; BCVA, best-corrected visual acuity; CMT, central macular thickness; VT, vessel tortuosity; VD, vessel density; DCP, deep capillary plexus; CC, choriocapillaris*.

**Table 4:** Most important characteristics of macular neovascularization in Group 1 and in Group 2.

**Parameter**	**Group 1 (VT <8.40)**	**Group 2 (VT > 8.40)**
Baseline BCVA	0.63	0.28
Baseline CMT	484	380
Final BCVA	0.24	0.43
Final CMT	362	324
Final atrophy	0%	30%
Speckled fluorescence (n. of quadrants)	1	1.6
MNV/leakage ratio	0.59	0.88
DCP VD	0.37	0.34
Number of injections	7.2	5.2

**Figure 4:**
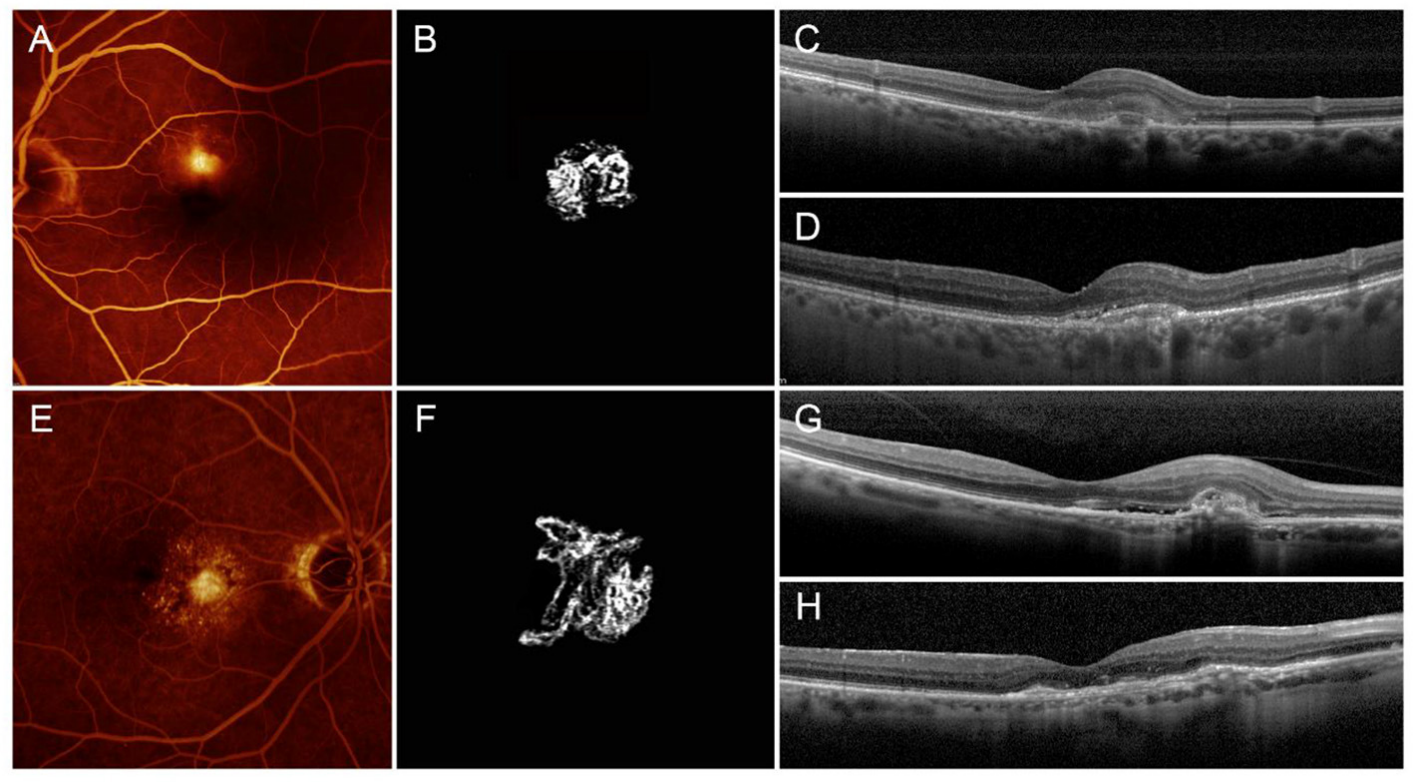
Low- and high-MNV VT neovascularizations. Low-MNV VT lesion is characterized by low-MNV/leakage ratio on FA **(A)**, scant SF, a poorly tortuous neovascular network on OCTA **(B)**, high-baseline exudation **(C)** and good anatomical and functional recovery after 1 year, with retention of outer retinal structure reflectivity properties **(D)**. High-MNV VT lesion is characterized by high-MNV/leakage ratio on FA **(E)**, involvement of high SF quadrants, a highly tortuous neovascular network on OCTA **(F)**, poor baseline exudation **(G)**, and bad anatomical and functional recovery after 1 year, with fibrotic alterations, and impairment of outer retinal structure reflectivity properties, together with backscattering phenomena **(H)**.

In summary, the main features of the two MNV subgroups are as follows: low-MNV VT lesions are characterized at the baseline by high exudation, extended blood-retinal barrier breakdown, and poor SF quadrants, leading to higher-baseline LogMAR BCVA, whereas the baseline features of high-MNV VT lesions are low exudation, less-pronounced blood-retinal barrier breakdown, high SF quadrants, and lower-starting LogMAR BCVA. The higher number of intravitreal injections, administered in a *pro re nata* setting over 1 year, together with less-affected DCP, leads to a better recovery of the BCVA. This recovery proves to be less pronounced in low-MNV VT lesions, compared with high-MNV VT lesions and is not associated with the onset of outer retinal atrophy, unlike high-MNV VT lesions, in which atrophy occurs in 30% of cases after 1 year (Figure 4).

Reproducibility and repeatability values of all the quantitative metrics varied overall from 0.91 to 0.97 and are fully reported in Table 5. The agreement between the two graders was very high for all the measurements, with overall ICC of 0.95 (range, 0.89–0.98).

**Table 5:** Reproducibility and repeatability values of OCTA quantitative parameters.

**Reproducibility and repeatability of quantitative parameters**		
**Parameter**	**Reproducibility**	**Repeatability**
Speckled Fluorescence (N. of quadrants)	0.95	0.96
Leakage area	0.94	0.95
MNV area	0.95	0.97
MNV/leakage ratio	0.92	0.91
VT MNV	0.95	0.93
VD SCP	0.94	0.93
VD DCP	0.92	0.91
VD CC	0.95	0.96

## Discussion

In the present study, we investigated the relationship between MNV OCTA-based VT values and MNV activity detected by FA and structural OCT.

Taking as a premise the traditional classification based on FA (15–18), we confirmed the presence of statistically significant differences between the different MNV types. In particular, type-2 MNV turned out to be more exudative (lower MNV/leakage ratio and greater CMT) compared with the mixed type, and type-1 MNV, although the final visual outcome proved comparable after the same number of intravitreal injections, had been administered in the three subtypes. Moreover, our quantitative MNV VT analysis failed to reveal any statistically significant differences between the three MNV types.

A different MNV classification based on MNV VT quantitative assessment performed by OCTA provided more information. In particular, the use of an MNV VT cutoff value of 8.40 identified two different MNV patterns, i.e., low VT and high VT, which had different baseline features and eventually led to different clinical and functional outcomes.

Low-VT MNV (MNV VT <8.40) corresponded to highly exudative lesions, with worse baseline BCVA and CMT, but with improved visual and anatomical outcomes after 1 year of treatment, following a significantly higher number of intravitreal injections (7.2 intravitreal injections). Conversely, high-VT MNV was characterized by less exudation—which explains the lower number of injections (5.2) administered in accordance with the *pro re nata* regimen—and by more frequent evolution toward atrophy and final visual function decline.

Interestingly, this kind of quantitative analysis also provided information about the anatomical constitution of the neovascular network. Indeed, the presence of a statistically significant correlation between MNV VT and MNV/leakage ratio, with the absence of correlations with the MNV area or leakage area alone, suggests that high-VT MNV, over than more perfused lesions, might represent better anatomically organized MNV, as regards to capillary walls. From this point of view, future studies assessing the relationship between quantitative imaging-based biomarkers and the histologic composition of the MNV might introduce intriguing new perspectives on the anatomical characterization of MNV lesions. With respect to the stability of MNV VT values over the follow-up, this was in accordance with a previous paper, showing that intravitreal treatments had no significant effect on this OCTA parameter (19).

The assessment of the MNV on the basis of VT suggests that a bias may occur in the ordinary therapeutic decision on the anti-VEGF administration, which is generally guided by the detection of exudative signs. Even though it appears less exudative, high-VT MNV may thus require a high number of anti-VEGF injections to be controlled.

Observing the FA findings, we detected a mismatch between leakage, taken as a sign of blood-retinal barrier breakdown, and the SF extension. Indeed, the low-VT MNV group revealed higher leakage and lower SF quadrants than the high-MNV VT group. SF may, therefore, represent a negative prognostic factor since its extension was significantly associated with clinically worse MNV. Furthermore, since the lower leakage and greater presence of SF were related to lower VD of the DCP, we might advance the hypothesis that SF is, in fact, a more reliable biomarker of pronounced vascular impairment than leakage.

A positive correlation was found between preserved DCP VD and visual acuity, in conformity with the findings of previous studies (6–10). In addition, we found that higher DCP VD correlated with increased exudation and leakage, suggesting that higher exudative phenomena might require a better preserved retinal vascular network. The higher DCP VD was possibly related to the vascular congestion secondary to the alterations in the perfusion distribution determined by the MNV, which may, in turn, have influenced the DCP as a result of both the mechanical burden and the release of growth factors and cytokines.

We are aware that our study has several substantial limitations. First of all, we restricted our analyses to type 1, type 2, and mixed MNV sub-forms that are readily detectableon OCTA, and, secondly, our follow-up was limited to just 12 months. The correlation we found on the basis of a VT cutoff of 8.40 may prove to be only valid for the specific MNV subtypes we considered and over a short-term follow-up. Hence, future studies with larger samples and longer follow-up would be needed to validate this new classification for clinical practice. Furthermore, OCTA can be conditioned by a number of image artifacts, which might have affected the analyses of the survey (20). In addition, the quantitative OCTA approach described requires supplementary software to analyze the images and, therefore, has limited relevance to clinical practice. A further shortcoming might concern the use of spectral-domain OCT technology instead of swept-source OCT, which is known to provide better images of deeper structures. However, it is worth pointing out that, although a swept source represents a step forward in OCTA analyses, spectral-domain technology remains a reliable way of detecting MNV, especially in the presence of naïve lesions (21). In addition, although we mainly interpreted SF as a sign of vascular impairment, we cannot exclude at all the possible contribution of drusen, focal RPE impairment, and other kinds of alterations to this FA finding. We have to acknowledge though that our pathogenetic hypotheses would need to be confirmed by histopathological validations. Just the same, while undoubtedly based on a limited number of eyes and a short follow-up, the present study provides new insights that may prove helpful in attempting to attain a better characterization of MNV secondary to AMD.

In conclusion, our study quantitatively assessed the relationship between MNV VT and its morpho-functional features. Based on our data, two different MNV subforms can be identified: low-VT MNV, which is more exudative at the baseline but less damaging to the outer retinal structures, and high-VT MNV, which is less exudative at the baseline but tends to lead to atrophic changes and functional deterioration. Further prospective studies are warranted to provide a more thorough investigation of the quantitative morpho-functional features characterizing MNV lesions so as to develop new models to optimize personalized treatment strategies. The quantitative approach adopted might form the basis of an artificial intelligence-based model, offering a better way to characterize MNV secondary to AMD.

## Data Availability Statement

The raw data supporting the conclusions of this article will be made available by the authors, without undue reservation.

## Ethics Statement

The studies involving human participants were reviewed and approved by Ethical Committee of the Vita-Salute San Raffaele University in Milan. The patients/participants provided their written informed consent to participate in this study.

## Author Contributions

AAr and EA: study design, data analysis, data interpretation, and manuscript draft. AB, AAm, and FBo: data collection, data analysis, manuscript revision. FBa and MB: data interpretation, manuscript revision. All authors contributed to the article and approved the submitted version.

## Conflict of Interest

The authors declare that the research was conducted in the absence of any commercial or financial relationships that could be construed as a potential conflict of interest.

## Publisher's Note

All claims expressed in this article are solely those of the authors and do not necessarily represent those of their affiliated organizations, or those of the publisher, the editors and the reviewers. Any product that may be evaluated in this article, or claim that may be made by its manufacturer, is not guaranteed or endorsed by the publisher.
